# Measuring resilience and assessing vulnerability of terrestrial ecosystems to climate change in South America

**DOI:** 10.1371/journal.pone.0194654

**Published:** 2018-03-19

**Authors:** Luciano J. S. Anjos, Peter Mann de Toledo

**Affiliations:** 1 Campus de Parauapebas, Universidade Federal Rural da Amazônia - UFRA, Parauapebas, Pará, Brazil; 2 Programa de Pós-Graduação em Ciências Ambientais - PPGCA, Instituto de Geociências, Universidade Federal do Pará - UFPA, Belém, Pará, Brazil; 3 Centro de Ciência do Sistema Terrestre - CCST, Instituto Nacional de Pesquisas Espaciais - INPE, São José dos Campos, São Paulo, Brazil; University of Oregon, UNITED STATES

## Abstract

Climate change has been identified as the primary threat to the integrity and functioning of ecosystems in this century, although there is still much uncertainty about its effects and the degree of vulnerability for different ecosystems to this threat. Here we propose a new methodological approach capable of measuring and mapping the resilience of terrestrial ecosystems at large scales based on their climatic niche. To do this, we used high spatial resolution remote sensing data and ecological niche modeling techniques to calculate and spatialize the resilience of three stable states of ecosystems in South America: forest, savanna, and grassland. Also, we evaluated the sensitivity of ecosystems to climate stress, the likelihood of exposure to non-analogous climatic conditions, and their respective adaptive capacities in the face of climate change. Our results indicate that forests, the most productive and biodiverse terrestrial ecosystems on the earth, are more vulnerable to climate change than savannas or grasslands. Forests showed less resistance to climate stress and a higher chance of exposure to non-analogous climatic conditions. If this scenario occurs, the forest ecosystems would have less chance of adaptation compared to savannas or grasslands because of their narrow climate niche. Therefore, we can conclude that a possible consolidation of non-analogous climatic conditions would lead to a loss of resilience in the forest ecosystem, significantly increasing the chance of a critical transition event to another stable state with a lower density of vegetation cover (e.g., savanna or grassland).

## Introduction

Empirical data show that in 2016 there was a drop in the temperature record [1]. It was also the driest year recorded for South America since observations began in 1900 [2]. Ongoing climate changes have been identified as the main threat to the integrity and functioning of terrestrial ecosystems in the 21st century [3]. In particular, projections for South America indicate that by the end of this century the continent is likely to be subjected to non-analogous climatic conditions, having little overlap with the current climate [4,5]. This represents a high risk to the extraordinary biodiversity on the continent due to the high climatic sensitivity of its ecosystems, which could lead to the compromise of a vast set of goods and services provided to humans [6,7].

Maintaining the structure and functioning of ecosystems over a broad-scale climate gradient range [8] suggests that each stable state of the ecosystem must be strictly adapted and evolved under a specific subset of abiotic conditions, enclosed in the multidimensional space of its climatic niche. Thus, under such a combination of conditions, the ecosystem is expected to have a higher capacity to absorb disturbances and recover the condition closer to the original state in the shortest time after a disturbance. Also, it is important to note that there is a strong correlation between ecosystem resilience and ecosystem structure.

The theory of ecological stability predicts that if changes to environmental conditions occur and a critical threshold of resilience is surpassed, catastrophic transitions between stable ecosystem states can be abruptly precipitated [9]. In this sense, it is crucial to measure and monitor ecosystem resilience, because this attribute plays a crucial role in mediating transitional events between different ecosystems [10]. Recent studies have been devoted to investigating and understanding the mechanisms behind critical transition events in tropical terrestrial ecosystems [11–14].

Despite recent theoretical and methodological advances, resilience is a concept that remains qualitative [15], and quantification at large scales can be challenging [16]. To overcome this limitation, we propose the methodological coupling of two significant bodies of theoretical knowledge in ecology: ecological stability theory [17] and ecological niche theory [18–20]. Some previous studies have already proposed measuring ecosystem resilience at large scales by calculating the likelihood of finding an ecosystem at a given level of precipitation [8,21,22]. However, none of the authors considered the robust theoretical framework and the recent methodological advances of ecological niche modeling [23,24]. With this approach, it is possible to construct spatially explicit models that measure the resilience of ecosystems through a metric of climate suitability, based on the multidimensional niche preferably occupied by them.

Overall, there are still many uncertainties about what effects climate change will have on the resilience and response of terrestrial ecosystems in structural and functional terms [25,26]. In this sense, it is fundamental to evaluate the intrinsic vulnerability of terrestrial ecosystems through a decomposition into three different axes: sensitivity, exposure, and adaptive capacity of the ecosystem [27]. Here, we propose to objectively estimate the comparative sensitivities of terrestrial ecosystems in South America from an analysis of ecosystem resistance to a gradual increase in climatic stress. Also, we will briefly assess the likelihood of exposure to unfavorable climatic conditions by identifying the propensity of each stable climate state to cope with non-analogous climate changes within or outside its optimal climatic niche, taking into account observed and simulated climate trends. Finally, to infer the adaptive capacity of ecosystems to changes in climate, we will assess the amount of climate variability that the ecosystem can tolerate without losing its structure and function.

## Materials and methods

### Study area

South America is located in the Neotropical biogeographic domain and has an area of almost 18 million km^2^ distributed among 12 countries. The continent has an extensive environmental heterogeneity, encompassing over 100 different ecoregions [28]. Among these are some of the most productive and megadiverse ecosystems on the planet, including forests (e.g., Amazon rainforest and Atlantic forest), savanna ecosystems (e.g., Cerrado, Beni, Gran-savanna), seasonally dry tropical forests (e.g., Caatinga, Chiquitano), and even deserts (e.g., Atacama).

The high variability can partially explain this diversity of ecosystems in latitudinal and altitudinal gradients, which directly influence the patterns of moisture and energy availability throughout the continent. Based on the climatic dataset of this study, we observed that average annual temperatures could vary between negative values at high altitudes (e.g., the Andes Mountain Range) to 30 °C in the low-altitude equatorial tropical zone. The annual range of temperature varies mainly as a function of latitude, being low near the equator, and can reach 18 °C in high latitudes. In some regions of the tropical zone, it can rain more than 6000 mm·year^-1^ with low seasonality throughout the year (e.g., Amazonian West), while other areas are exceptionally arid, with a marked dry season throughout the year and less than 100 mm·year^-1^ of precipitation.

### Vegetational cover data and determination of stable states

To define the terrestrial ecosystems of South America as stable states, we used the variable called tree cover for the year 2001, from the Moderate Resolution Imaging Spectroradiometer (MODIS) satellite sensor. This variable describes the percentage of vegetation cover varying between zero and 100% [29] and can be interpreted as the density or abundance of trees. The transition limits between ecosystem’s stable states were inferred from the frequency distribution of the tree cover [8], which presented a trimodal distribution. Each distribution mode comprises a stable state of a terrestrial ecological system. The grasslands had zero to 5% tree cover; savannas had 5 to 60%, and forests had values above 60%. The original dataset, spanning all of South America, was resampled from a 500-m to a 6-km spatial resolution.

Due to the effects of historical changes in land use, mistaken pixel classifications occurred among the three ecosystem classes. Notably, the forest ecosystem (with tree cover between 60 and 100%) was the most sensitive to spectral and radiometric changes following the intrusion of alternative land uses (e.g., selective logging, monocultures, or pastures). To minimize such classification bias, and thus to match the original distribution of the forest ecosystem as closely as possible, we evaluated the classification accuracy by comparing the tree cover with a census database of high-resolution land cover [30]. Whenever a pixel was classified with a tree cover percentage of less than 60% but classified as forest based on the consensus dataset, the pixel was reclassified to forest.

### Presence-absence data for ecological niche modeling

After correction of land use classification inaccuracies, the raster database was converted to a presence-absence binary, where each map pixel was converted to a point in vector format. In total, *n* = 37763 samples were generated for South America, of which 53% were classified as savanna; 38% as forest; and 9% as grassland. These ecosystems are mutually exclusive and geographic substitutes, so when a particular type of ecosystem was recorded as present (1) for a given area, the others were automatically recorded as absent (0) in that same area. These presence-absence data were used as an input in the ecological niche modeling procedure described below. Each point was assigned a geo-referenced signature with longitude and latitude information.

### Climatic variables and selection of bioclimatic predictors

For this work, the climatic conditions of South America were defined along two principal axes: availability of moisture (1) and energy (2). At the scale used for this study, these are considered the primary predictors of climate response patterns in terrestrial ecosystems [31,32].

To describe the pattern of moisture availability, we used current precipitation data for South America from the climatic dataset CHPclim (v.1.0), produced by the Climate Hazards Group’s Precipitation Climatology. It is a combination of satellite data, physiographic indicators, and standard in situ climatological data, with data available from 1980 to 2009. The final product is a monthly global climatic precipitation measure with a spatial resolution of 0.05° (~6 km) [33]. This database was copied from the address 'http://chg.geog.ucsb.edu/data/CHPclim/' in ‘tiff’ format and then cut to the region of interest. CHPclim has a critical comparative advantage over other precipitation climatic bases available for South America (e.g., WorldClim): It has a better fit for regions with low densities of meteorological stations and high variability of precipitation, such as the Amazon, and responds satisfactorily to complex terrain, such as in the Andes Mountain Range [33].

To describe energy availability across the continent, we used the WorldClim temperature database [34], which consists of a climatological average between the years 1960 and 1990, with data from stations around the globe. The spatial resolution of the dataset is 0.041° (~ 5 km) and was copied from 'http://www.worldclim.org/version1' in ‘tiff’ format. Although it has the same limitation of a low density of meteorological stations for the Neotropical region, the variable in question does not present significant variability near the equator, so that the quality of the observed data is not compromised. Finally, we selected four bioclimatic predictors related to energy availability and moisture, which originate from the ecophysiological point of view of ecosystems: (1) annual cumulative precipitation (ACP), (2) precipitation seasonality coefficient (PSC), (3) average annual temperature (AAT), and (4) annual range of temperature (ART).

### Modeling the climatic niche of ecosystems

Ecological niche modeling finds strong support in ecological niche theory [35], which predicts that each organism has a multidimensional environmental space with optimal conditions for its survival, growth, and reproduction [36–39]. The various modeling methods quantify the relationships between occurrences (presence/absence) and environmental predictors to delimit and adjust the multidimensional environmental niche. The result is the quantification and spatialization of the suitability of the environment for the modeled ecosystem.

To model the distribution of ecosystems, we used the biomod2 package implemented in R software [40]. Distribution models were calibrated using presence-absence data from each ecosystem and climate predictors for the South American continent. We have adopted the ensemble strategy that emphasizes the most consensus in predictions among different modeling methods [23,41], thus minimizing the effect of uncertainties on model prediction [42]. The models were run using 10 different methods: Bioclim (SRE), Classification Tree Analysis (CTA) [43], Maxent [44,45], Random Forest (RF) [46], Generalized Linear Models (GLM) [47], Generalized Additive Models (GAM) [48], Generalized Boosted Regression Models (GBM) [49], Function Discriminant Analysis (FDA) [50], Artificial Neural Networks (ANN) [50], and Multiple Additive Regression Splines (MARS) [51].

For each method, ten replicates with 75% and 25% partitions were run for training and testing, respectively. Quality of the models produced by the different methods was evaluated using True Skill Statistics (TSS) and Receiver Operating Characteristic (ROC) metrics (S1 Table). We calculated the contribution of each bioclimatic predictor to build and explain the patterns of response variable which varies between zero (lower importance) to 1 (higher importance) (S1 Table). The models selected to compose the ensemble were those with the best TSS, which measures combined sensitivity and specificity of the model [52]. For the threshold effect, only the models with TSS values equal to or greater than 0.7 were considered for inclusion in the ensemble. The consensus distribution model was then obtained as the arithmetic average taken for the selected models [53].

### Measuring and mapping the resilience of terrestrial ecosystems

Here we present an alternative interpretation for the climate suitability map produced from ecological niche modeling. For the first time, a methodological convergence between predictions of ecological stability and ecological niche theory is explicitly suggested. The phenotypic adaptation of an ecosystem to a given set of climatic conditions has a direct and robust relationship with the resilience of the ecosystem itself. In this sense, the ecosystem is expected to lose resilience as it moves away from the optimal climatic conditions of its niche. Therefore, the product of niche modeling can be interpreted as a continuous and objective measure of resilience, ranging from zero (minimum resilience) to 1000 (maximum resilience) for terrestrial ecosystems in South America.

### Sensitivity of terrestrial ecosystems to climate stress

We assume that climate stress increases in ecosystems as they move away from their optimal niche. Since the climate suitability gradient, predicted by niche modeling, can be interpreted as an approximation of the fundamental ecosystem niche, we estimate climate stress from a simple inversion of the gradient as predicted by the models. Thus, a band with low climatic suitability is assigned a high level of climatic stress and vice versa.

Using this climate stress vector (x) as the predictor of the relative frequency of observations for each ecosystem (y), we fit an exponential model according to the equation below:

$$y=a^{bx}$$ (1)

where a and b are the estimated coefficients of the exponential model. The objective was to use the coefficient b, which defines the curve fit of the exponential model to the observed data, as an indicator of the ecosystem resistance to climatic stress. In other words, if a small increment of climatic stress significant changes the ecosystem, we can assume that the ecosystem has low resistance to climatic stress, and consequently a higher value of the coefficient b. Conversely, resistant ecosystems will have a lower value of the coefficient b.

### Exposure of ecosystems to non-analogous climatic conditions and their respective adaptive capacities

In a two-dimensional climatic space representing the availability of moisture and energy present in South America, we evaluated the propensity of ecosystems for exposure to non-analogous climatic conditions and their respective adaptive capacities based on the climate space occupied by each ecosystem and its respective observed resilience gradient. We have taken as a reference the observed current climate trends [2,54,55] and simulations of future climate scenarios [5,56,57] to assess the likely progression of climate changes in South America. In general, both observed and simulated data point to an increase in climatic seasonality associated with increased aridity and temperatures for South America. We, therefore, delimited a polygon for the bidimensional climate space to indicate the likely orientation climate changes on a continental scale. Observed ecosystem resilience data was then compared with the polygon to determine which ecosystems would be most abruptly exposed to unfavorable climatic conditions. In the same sense, it was also possible to observe the response width of ecosystem resilience and, in this way, infer the capacity to adapt to a new climatic reality.

## Results

### Mapping ecosystem resilience

**Fig 1:**
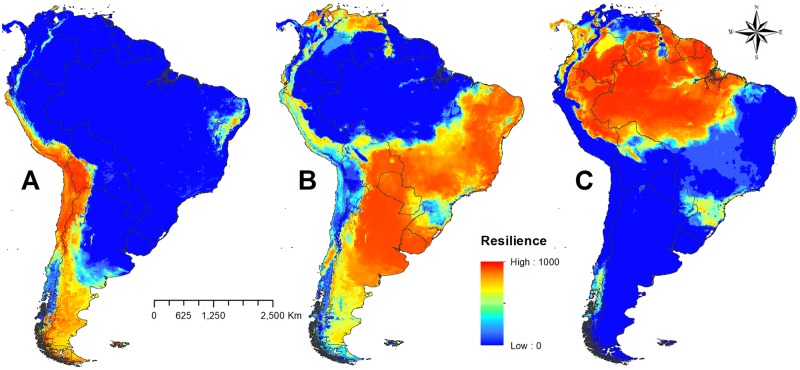
Ecosystem resilience based on the climatic niche projected under geographic space. A Grasslands; B Savannas; C Forest. High values (~1000) indicate a high recovery capacity after a disturbance, whereas low values (~zero) mean slower recovery capacity after a disturbance.

Here we present a continuous metric of spatially explicit ecosystem resilience, based on the climatic niche to which each ecosystem is adapted (Fig 1). Grasslands have high resilience at high altitudes and arid environments, such as in the Andes, the Atacama Desert, and Patagonia. Savannas are widely distributed throughout the continent and exhibit high resilience along the continental diagonal polygon as well as to the north of South America. Forests have good resilience near the equator, covering almost entirely the Amazon basin to the foothills of the Andes. Forests also occupy a narrow strip following the Atlantic coast (Atlantic Forest) and the eastern region lying near latitude 50° (Araucaria forest). All of these areas show a lower level of resilience than do the forests of the Amazon basin. In the southwestern portion of the continent, the model predicted the presence of a vertical band of temperate forest (Valdivia).

There is a spatially structured pattern of loss of resilience toward the transition zones between the three ecosystems. Models based on the climatic niche of ecosystems were highly sensitive in detecting that ecotone zones tend to have a lower resilience value than do areas in an optimal climate niche. This pattern reinforces the idea of geographic ecosystem replacement caused by transitional events through the loss of resilience through geographic space and climatic gradient.

### Sensitivity of terrestrial ecosystems to climate stress gradient

**Fig 2:**
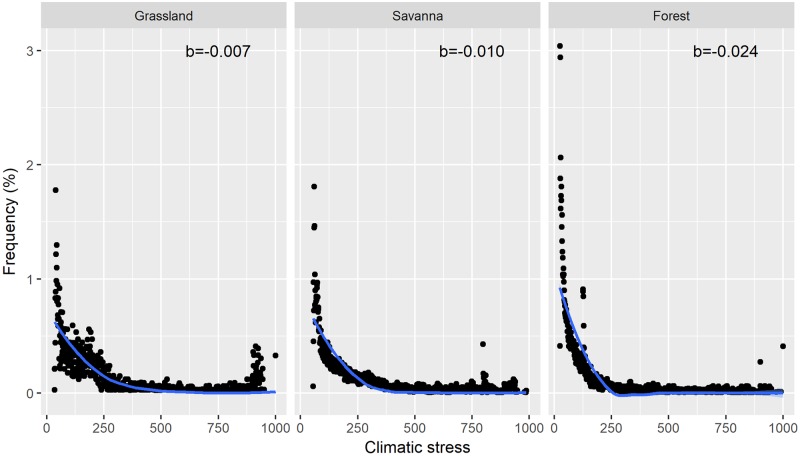
Model of ecosystem responses to a climate stress gradient based on the prediction of relative frequencies (%). The parameters of the exponential model indicate that there are differences regarding resistance to climatic stress among the three ecosystems. Grassland n = 598; Savanna n = 889; Forest n = 891.

In general, the three ecosystems presented similar statistical behaviors, with a decrease in their relative frequencies accompanying a gradual increase in climatic stress (Fig 2). However, there are significant differences concerning the forms of the response curves observed for the three ecosystems. For savannas and grasslands, the distribution of observations was more uniform, while forests account for almost 80% of the observations in the range of the lowest climatic stress (close to the climatic optimum). Because of this, savannas and grasslands have a smoother response curve as climate stress increases than do forests. This pattern was also reflected in the value of the exponential model parameter, with forests (b = -0.024) having a value almost double those for grasslands (b = -0.010) and savannas (b = -0.007), indicating that savannas and grasslands have better resistance to climatic stress than do forests.

### Propensity for exposure to unfavorable climatic conditions

**Fig 3:**
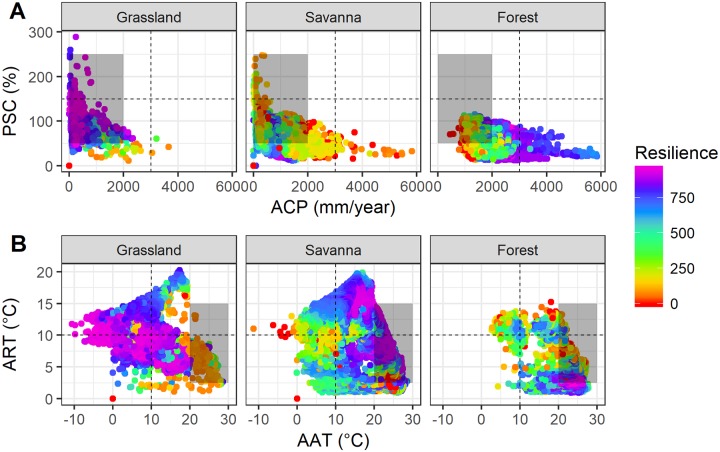
Observed patterns between climatic variation and ecosystem resilience. In the two-dimensional gradient of moisture availability (A) and energy availability gradient (B), if ecosystems are exposed to non-analogous climatic conditions (gray polygon), it is possible to assess what the impacts would be and whether they are likely to adapt to climate change. Forest observations: n = 14,466 samples—38.30%; Savana: n = 19,923 samples- 52.75%; Grassland: n = 3,374 samples—8.93%.

The moisture availability gradient showed a strong interaction with climate resilience. Forests present high resilience to rainfall above 2000 mm·year^-1^ with a relatively low seasonality (<100%) during the year (Fig 3). Savannas are adapted to a precipitation range between 500 and 1800 mm·year^-1^, so that resilience tends to decrease with increasing seasonality of precipitation and annual rainfall volumes. In contrast, grasslands show high resilience in the gradient range where a more arid climate prevails, with low rainfall volumes and high rainfall seasonality throughout the year. In this sense, if the observed trends are assessed alongside the simulated future climate scenarios, both savannas and grasslands would be favored, while forests would inevitably lose resilience due to moisture reduction.

For the energy availability gradient, forests are adapted at high levels of resilience to a narrow range of high average annual temperatures (between 20–28 °C), but tolerate only low variability throughout the year (between 0–5 °C·year^-1^). Outside of this range, there was a drastic loss in ecosystem resilience. On the other hand, savannas have high resilience at average annual temperatures above 10 °C, also supporting temperatures above 25 °C, while their resilience tends to decrease at temperatures below 10 °C. Savannas also persist in places with considerable variation in temperature throughout the year, with ranges up to 20 °C. Grasslands are adapted for temperatures in a wide range, from negative to 20 °C average annual temperatures, and with a large annual thermal amplitude (5 to 20 °C·year^-1^). The observed and simulated average temperature trends indicate abrupt increases, as delimited by the gray polygon, which would favor forest ecosystems, provided there is no increase in the annual temperature range. If there is an increase in annual temperature variability, the scenario would be more favorable to savannas at the expense of forests, which would probably lose resilience.

### Adaptive capacity of terrestrial ecosystems

Forests and savannas have strong niche-specific adaptive responses to moisture availability persisting over a specific range of the moisture gradient (Fig 3). This suggests a low adaptive capacity to new conditions outside the optimum climatic niche for forest and savanna ecosystems. On the other hand, grasslands show high levels of resilience to a wide range of moisture availability. The pattern is further enhanced by the seasonal axis of precipitation, with grasslands maintaining high levels of resilience for a wide range of moisture availability. The most significant adaptive constraint regarding grassland resilience is regions with rainfall volumes over 2000 mm·year^-1^.

Savannas and grasslands would have higher adaptive capacities to changing temperature gradients because they show high resilience at a wide range of temperature combinations. The forest ecosystem, however, has a relatively narrow climatic niche and demonstrates high resilience only for high temperatures with low annual variability. This suggests that savannas and grasslands have a higher adaptive capacity to changes in the energy availability than do forests, which have a narrow climatic niche.

## Discussion

In the context of robust transformations induced by climate change, the resilience estimated from the climatic niche emerges as an alternative to the traditional metrics of biological diversity (such as richness or species composition). This new metric can be interpreted as a measure of ecosystem health [58], and may be useful in the design and implementation of conservation strategies at different spatial scales. For example, ecosystem resilience could be monitored in real time to predict catastrophic transitional events between stable states as a function of observed or simulated climate changes [13]. This method is already available, since remote sensing data are widely available at high spatial, spectral, and temporal resolutions [59].

Another potential application would be in actions to support the restoration of degraded ecosystems, considering that the success of an ecological restoration process is directly linked to the ecosystem resilience of the area to be restored. In other words, recovering a forest in an area with low resilience (for this type of ecosystem) would be more costly regarding resources and time, if not infeasible, which could jeopardize successful project implementation.

Our results indicate that the terrestrial ecosystems of South America are sensitive to the expected gradual increases in climatic stress, since the relative frequency of ecosystems decrease as conditions become marginal for the climate niche of the ecosystem in question. However, there are differences in the form of responses between different vegetation classes. When the exponential model parameters were compared between ecosystems, forests presented the least resistance to climate stress, indicating a higher sensitivity intrinsic to changes in climate. These results suggest that forests are more sensitive to climate variability [11,60], mainly due to decreasing availability of moisture [61–63]. This result highlights the fragility of these ecosystems to climate change since vulnerability is positively correlated with sensitivity [27].

The response curve for the stable state of forests also indicates that an incremental change in the climatic stress gradient could trigger large-scale transformations, particularly if a rupture threshold is reached [64,65]. This factor may be critical in the short term, with the accelerated levels of transformation currently being experienced, so it is critical to the identify thresholds for rapid forest decline. Even so, it may take decades for forests to restore the services they provide [58]. In contrast, a larger amount of climate stress would be required for savannas or grasslands to become more susceptible to large-scale transformations. It is possible to explain their higher tolerance to climatic stress due to better adaptation to water deficit and the fire effect on biota [66,67].

However, it is important to note that when forests are subjected to some degree of climatic stress, particularly to moisture deficit, their capacity to retain CO_2_ in biomass is compromised [68–71]. The reduction of moisture availability throughout the year affects the floristic composition of forest species [72] and is also associated with high tree mortality rates [73,74]. Hydric stress, combined with factors such as fire, can lead to species loss and biodiversity erosion [75,76]. Furthermore, changes in land use at large scales, e.g., deforestation or selective logging, have affected biophysical climatic parameters such as air temperature [77], as well as the precipitation regime [78,79]. Since temperature is an essential predictor of the maintenance of structure and function of terrestrial ecosystems, these bioclimatic temperature variations are expected to directly and negatively affect forest resilience.

Our results indicate that forests in South America are more likely to face unfavorable climatic conditions in the near future than are savannas and grasslands, which have a lower percentage of vegetation cover. Forest ecosystems display greater vulnerability due to their higher probability of exposure to non-analogous climates [27]. Empirical evidence shows that forests in border areas of the Amazon southwest have presented slower biomass recovery after extreme drought events [80,81], suggesting that they have recently declined in resilience.

On the other hand, these new climatic conditions are likely to favor other ecosystem types that display increasing resilience. In this way, the dynamics of resilience within an ecological system with multistability are closely related to transition events between stable states and depend directly on the orientation of change, thus favoring a particular stable state. For example, at the end of the Upper Miocene, there was a reduction in global temperature, and grassland ecosystems dominated most of South America [82]. However, in the Eocene, with higher temperatures and precipitation, forests thrived to the detriment of grasslands and savannas [83].

Tropical forests are known to have a narrow thermal tolerance [84,85], evidence of their higher vulnerability to climate change, representing a low adaptive capacity compared to other ecosystems that can maintain high resilience values under a wide range of climatic conditions. Moreover, in theory, for an adaptation event to occur, the adaptive strategy should be within the limits of the diversity of responses, i.e., phenotypic plasticity, displayed in the current climate. Outside the boundaries of the climatic niche, there would be slow adaptation, and the ecosystem would succumb to a loss of structure and function until it reached a point of rupture or another stable state.

Our results indicate that, in this sense, savannas demonstrate an advantage over forests. Savannas are successful in a wide range of moisture and temperature availability and tolerate strong seasonal variations. Savannas can, therefore, maintain a steady tree cover state despite climatic variation, which confers a tremendous adaptive potential to the ecosystem if it is exposed to non-analogous climatic conditions.

There are no predicted energy restrictions in the near future, since all simulated projections point to an increase in temperature, regardless of the emissions scenario [86]. An increase in atmospheric CO_2_ concentration could favor forests by increasing the availability of CO_2_ as a resource [87,88]. However, the most critical factor for forest persistence is the widespread need for moisture [62,89,90]. In contrast to the hypothesis that forests are resilient to climate change [87], our results suggest that, due to the close adaptive relationship of ecophysiological and evolutionary patterns between terrestrial ecosystems and climate, the high values of resiliency to changing climatic conditions can only be maintained at the conditions of the optimum climatic niche occupied by the ecosystem. This dependence would thus be reflected in ecosystem responses following a disturbance event [91].

## Conclusions

Our models, with strong support in niche theory and ecological stability, have shown a significant sensitivity and may be useful in several practical applications within the science of conservation, including in assessing the vulnerability of ecosystems to climate change. We found some worrying evidence that South American forest ecosystems are intrinsically more vulnerable to climate change than other ecosystems. Ongoing climate change can accelerate the loss of ecosystem resilience by promoting erosion of forest biodiversity and leading to another stable state with a lower density of vegetation cover.

## Supporting information

S1 Table Importance metrics of the predictors to build the ecological niche models and evaluation metrics by different methods for each ecosystem. Average values were calculated utilizing 10 replicates, as well as the evaluation metrics.
(PDF)
