# Supplementary material for: Measuring resilience and assessing vulnerability of terrestrial ecosystems to climate change in South America
Source: PLoS One. 2018 Mar 19;13(3):e0194654. doi: 10.1371/journal.pone.0194654 (PMC5858834; doi:10.1371/journal.pone.0194654)
Supplement: S1 Table — Average values were calculated utilizing 10 replicates, as well as the evaluation metrics. (PDF) [file pone.0194654.s001.pdf]

Supporting Information

S1A Table

**Grassland**	Variable importance				Metrics for validation	
**Modeling method**	**Precipitation seasonality coefficient**	**Annual cumulative precipitation**	**Annual temperature range**	**Annual average temperature**	**TSS***	**ROC***
CTA	0.04	0.5	0.26	0.57	0.8	0.94
Maxent	0.08	0.2	0.16	0.5	0.75	0.94
GBM	0.02	0.49	0.07	0.32	0.79	0.95
RF	0.17	0.49	0.32	0.44	0.97	0.99
GLM	0.05	0.45	0.01	0.39	0.74	0.93
GAM	0.04	0.29	0.08	0.53	0.76	0.94
FDA	0.09	0.22	0.08	0.39	0.75	0.92
SRE	0.24	0.4	0.39	0.25	0.69	0.8
ANN	0.08	0.47	0.02	0.34	0.77	0.94
MARS	0.02	0.3	0.08	0.52	0.76	0.93
**Mean (std)**	**0.08 (0.06)**	**0.38 (0.11)**	**0.15 (0.13)**	**0.42 (0.10)**		

S1B Table

**Savanna**	Variable importance				Metrics for validation	
**Modeling method**	**Precipitation seasonality coefficient**	**Annual cumulative precipitation**	**Annual temperature range**	**Annual average temperature**	**TSS***	**ROC***
CTA	0.05	0.72	0.46	0.24	0.74	0.92
Maxent	0.03	0.41	0.44	0.21	0.63	0.88
GBM	0.01	0.57	0.41	0.09	0.68	0.92
RF	0.14	0.69	0.47	0.31	0.95	0.99
GLM	0.01	0.57	0.4	0.1	0.6	0.85
GAM	0.02	0.43	0.46	0.08	0.64	0.9
FDA	0.01	0.51	0.24	0.06	0.61	0.88
SRE	0.21	0.51	0.18	0.19	0.62	0.75
ANN	0.07	0.67	0.39	0.12	0.68	0.91
MARS	0.01	0.47	0.41	0.06	0.63	0.89
**Mean (std)**	**0.05 (0.06)**	**0.55 (0.10)**	**0.39 (0.09)**	**0.15 (0.08)**		

S1C Table

**Forest**	Variable importance				Metrics for validation	
**Modeling method**	**Precipitation seasonality coefficient**	**Annual cumulative precipitation**	**Annual temperature range**	**Annual average temperature**	**TSS***	**ROC***
CTA	0.08	0.71	0.3	0.09	0.85	0.96
Maxent	0.11	0.34	0.41	0.23	0.77	0.95
GBM	0.01	0.58	0.33	0.02	0.82	0.97
RF	0.1	0.64	0.36	0.22	0.96	0.99
GLM	0.02	0.65	0.23	0.03	0.77	0.95
GAM	0.03	0.52	0.43	0.06	0.81	0.96
FDA	0	0.54	0.18	0.06	0.78	0.95
SRE	0.14	0.56	0.25	0.26	0.64	0.82
ANN	0.08	0.74	0.25	0.06	0.81	0.96
MARS	0	0.53	0.36	0.04	0.8	0.96
**Mean (Std)**	**0.05 (0.05)**	**0.58 (0.11)**	**0.31 (0.08)**	**0.11 (0.09)**		

S1 Table. Importance metrics of the predictors to build the ecological niche models and evaluation metrics by different methods. Average values were calculated utilizing 10 replicates, as well as the evaluation metrics.
